# Application of a classroom-based positive psychology education course for Chinese medical students to increase their psychological well-being: a pilot study

**DOI:** 10.1186/s12909-020-02232-z

**Published:** 2020-09-22

**Authors:** Xiao-Qin Zhang, Bao-Shuai Zhang, Meng-Die Wang

**Affiliations:** School of Medicine, South China University of Technology (SCUT), Guangzhou, 510006 People’s Republic of China

**Keywords:** Positive psychology, Chinese medical students, Psychological well-being

## Abstract

**Background:**

Anxiety and depression have been increasing among Chinese medical students. The psychological well-being of Chinese medical students has become a critical focus of attention for the medical education community. Increasing evidence shows that positive psychology interventions can be effective in the enhancement of psychological well-being, and may help to prevent depressive symptoms in university students. In the present study, we aimed to explore the potential effect of positive psychology education on improving the mental health of Chinese medical students.

**Methods:**

An 8-week classroom-based positive psychology intervention workshop, which was established as an elective course embedded in the regular school curriculum, was conducted at the School of Medicine, South China University of Technology (SCUT), China. Undergraduate medical students of the institute in year-2 or year-3 of academic study participated in this training course voluntarily. The participants’ self-reported data on the hope scale, life satisfaction scale, subjective happiness scale, and depression and anxiety scale were collected and analyzed at pre-course (*n* = 61) and post-course (*n* = 49) times. The investigation was also validated with an independent cohort of students who enrolled in the course in the year following the preliminary study.

**Results:**

The analyses showed that the psychological well-being of the participants were improved after the intervention. Their mean scores on the hope scale, life satisfaction scale and subjective happiness scale were significantly improved (*P* < 0.05), while their symptom levels of depression and anxiety were significantly reduced (*P* < 0.01). A similar trend was observed in the validation cohort.

**Conclusions:**

These preliminary findings suggest that positive psychology education holds promise for improving psychological well-being among Chinese medical students. Further investigations with larger and well-controlled sample cohorts may yield more convincing and reliable results.

## Background

Training in medicine is highly emotionally and physically demanding, which has led to prevalent anxiety, depression and distress among medical students worldwide [1–8].

This is especially true for Chinese medical students and young trainees due to the heavy academic pressure and deteriorating doctor-patient relationship in China [8–12]. It has been well known that medical school in China is entered from high school, whereas in the United States and many other countries it is entered after an undergraduate degree, which means that Chinese medical students need to complete general education study as well as professional study within a shorter time [13]. Meanwhile, increasing health demand in China driven by the improving economy during the past decade has led to an excessive burden on Chinese doctors, which results in increasing conflicts between patients and doctors [9–12, 14–17]. It has been reported that at least one-third of Chinese doctors have experienced conflicts with patients [8]. The increased emotional and physical exhaustion, together with the depersonalization and a low sense of personal achievement have contributed greatly to the increased levels of stress symptoms among Chinese doctors, a phenomenon that has also spread to medical students. It has been reported that over 60% of Chinese medical students suffer different degrees of depressive symptoms and suicide attempts [18, 19]. Compared with the general population, they are more susceptible to stress, burning out and anxiety, and have less career satisfaction and self-esteem as compared with their counterparts [18–21]. The psychological well-being of Chinese medical students has become a matter of national concern and needs urgent intervention.

To deal with these situations, a series of strategies have been adopted by multiple levels of healthcare administration institutes in China. For example, psychology education has been increasingly emphasized in universities [22, 23], and an increasing number of school consulting offices have been established [24]. While these interventions provide helpful psychological support for the few students with existing psychological disorders, they fail to serve the remaining large population of general students who do not have a diagnosed disorder yet [24]. Moreover, these psychology interventions were more pre-occupied with weakness and disorders, such as the depression, anxiety and stress, but seldom develop the virtues and character strength that facilitate individual thriving, such as resilience and grit, which are important for the personal growth of young people [25, 26].

An increasing number of studies have shown that positive psychology interventions can enhance psychological well-being and alleviate depressive symptoms among students and the general public [27, 28]. Positive psychology is a new branch of traditional psychology, that focuses on optimal human functioning and strength instead of the weakness and illness that characterize traditional psychology [29–31]. It focuses on the strengths and virtues that facilitate individual thriving through various ways, such as counting blessings, practicing kindness, setting personal goals, expressing gratitude and using personal strength [27, 28]. The incorporation of positive psychology into school education can significantly increase the resilience and psychological well-being of students and finally improve their academic accomplishments [27, 28, 32–35]. Given the unprecedented level of anxiety and depression in Chinese medical students and the lack of sufficient mental health care in schools [36], there is a great need to investigate how insights from the field of positive psychology can help medical students flourish in both their professional studies and personal lives. However, an investigation of positive psychology interventions for Chinese medical students to enhance their psychological well-being has not been reported thus far.

In the present study, we aimed to examine the effects of a classroom-based positive psychology intervention workshop on improving the psychological well-being of undergraduate medical students in our institute. The participants’ scores on the hope trait scale, life satisfaction scale, subjective happiness scale, and depression and anxiety scale were measured before and after the training course, respectively. Our hypothesis was that the classroom-based positive psychology intervention would increase the psychological well-being and reduce the depressive and anxiety symptoms of medical students.

## Methods

### Study design

The positive psychology intervention was established as a classroom-based elective course, which was embedded in the regular school curriculum and open to medical students at the School of Medicine, South China University of Technology (SCUT), Guangzhou, China. The course was open to year-2 and year − 3 students only. The attendees who voluntarily consented to participate in the research were asked to complete the questionnaires before and 1 week after the training course, respectively. All questionnaires were anonymous, with the exception of academic year, gender and age, to avoid stigmatizing participants and to obtain honest answers as much as possible. No compensation was provided for participation. To test the effects of the intervention, an identical set of interventions and surveys was performed on an independent cohort of students who attended the course voluntarily in the year following the preliminary study. None of the participants reported any previous experience with positive psychology intervention.

### Participants

A total of 61 undergraduate medical students in year-2 or year-3 of academic study attended the training course voluntarily in the preliminary study. Only participants who completed the entire course study, assignments, and pre- and post-course questionnaires were included in the analysis. According to these inclusion criteria, of the initial 61 participants recruited to the study, 12 dropped out of the program, giving an attrition rate of 19.7% (12/61). For these 12 students who discontinued the study, their common reasons were that they were too busy to finish the assignments or return the post-course questionnaire. For the 49 participants, their ages ranged from 17 to 22 years (mean = 19.5, SD = 0.94). For the validation cohort, 52 students participated in the course initially, and 46 of the participants who fulfilled the above inclusion criteria were included in the current analysis. Six of them dropped out of the program due to time limitations. The detailed socio-demographic characteristics of both participant cohorts are described in Table 1.

**Table 1:** Socio-demographic characteristics of the medical students enrolled in the study

Variables	Preliminary set		Validation set	
	Pre-course ***N*** = 61	Post-course ***N*** = 49^a^	Pre-course ***N*** = 52	Post-course ***N*** = 46^b^
**Age (range, M, SD)**	17–22, 19.4, 0.92	17–22, 19.5, 0.94	17–21, 18.3, 0.79 0.79	17–21; 18.5, 0.78
**Academic year**				
Year 2	36 (59%)	31 (63.3%)	52	46 (100%)
Year 3	25 (41%)	18 (36.7%)	0	0
**Gender**				
Female	43 (70%)	36 (73.5%)	35 (67.3%)	31 (67.4%)
Male	18 (30%)	13 (26.5%)	17 (32.7%)	15 (32.6%)

*Abbreviations*: *M* mean, *SD* standard deviation

^a^12 students discontinued the study, ^b^6 students discontinued the study

### Procedures

The students who participated in the course were first invited to complete a packet of questionnaires (as detailed in the following ***Measures*** section and Appendix 1-5) prior to the commencement of the training course. They then received the training and finished the related assignments for 8 continuous weeks (as detailed in the following ***Interventions*** section). Finally, in the week following the completion of the course, they were given an identical packet of questionnaires to complete. The pre- and post-course questionnaires were then compared and analyzed. At the same time, an independent survey question would be given in the post-course questionnaire by asking “Overall, what do you think of this project?”. Participants were asked to rate their response with a four-item Likert-type scale including “No useful at all”, “A little useful”, “Useful” and “Very useful”.

### Interventions

The intervention was set as a 1.5-h class once a week and lasted for 8 continuous weeks. The protocol of the intervention, which is detailed in Appendix 6, was derived from Dr. Martin Seligman’s theory of PERMA (Positive emotion, Engagement, Relationship, Meaning, Accomplishment) [37] with slight modifications. Briefly, in each weekly class, a different topic related to the cultivation of PERMA was discussed, such as the cultivation of positive emotional states (e.g., gratitude and appreciation), the cultivation of intrinsic motivation through “flow”, and learning to be in harmony with bad moods by highlighting the meaning of life. Additionally, multiple topics related to medical professionals (e.g., doctor-patient relationships) were introduced and discussed in class to guide the students to find the thinking traps using positive psychology theory. Multiple out-of-class exercises were also assigned, such as writing down good things and identifying key character strengths. One single teacher led and completed the entire intervention, and the teacher had been trained to be qualified before the class at the Center for Positive Psychology and Engineering Psychology, School of Social Sciences at Tsinghua University, Beijing, China.

### Measures

The following five scales were used to measure the psychological status of the participants before and after the training: the trait hope scale, life satisfaction scale, subjective happiness scale and depression and anxiety scale (Appendix 1–5). All these questionnaires were first translated from English into Chinese by one of the authors who was fluent in both Chinese and English. The translations were then checked by another author of the study to ensure the consistency with the original meaning of the scale items. No other adaptations to these scales were made.

#### The trait hope scale

The hope trait was measured by using the 12-item Trait Hope Scale [38, 39] (Appendix 1). This questionnaire asks respondents to rate their agreement with 12 statements related to hope on an 8-point Likert-type scale ranging from 1 (definitely false) to 8 (definitely true) (Cronbach’s α = 0.85). A sample item of the scale is “I can think of many ways to get the things that are important to me”. Among the scale items, 4 items (item 2, 9, 10 and 12) measure the goal-directed energy (also called agency thoughts), and 4 items (item 1, 4, 6 and 8 in the questionnaire) measure the plans to meet goals (also called pathway thoughts). The total trait hope scale score is derived by summing the four agency and the four pathway items. The possible range is 8–64, with higher scores reflecting higher levels of hope.

#### Life satisfaction scale

The life satisfaction was measured using the 5-item satisfaction scale [40, 41] (Appendix 2). The scale utilizes a 7-point Likert-type response scale ranging from 1 (strongly disagree) to 7 (strongly agree) (Cronbach’s α = 0.86). A sample item of this scale is “In most ways, my life is close to ideal”. The total score is derived by summing all 5 items. The possible range is 5–35, with a score of 20 representing a neutral point, 5–9 indicating extreme dissatisfaction with life and 31–35 indicating extreme satisfaction.

#### Subjective happiness scale

The subjective happiness scale is a 4-item scale of global subjective happiness [42, 43] (Appendix 3). Each item is rated on a 7-point Likert-type scale ranging from 1 (strongly disagree) to 7 (strongly agree) (Cronbach’s α = 0.89). Amongst, two of the items ask respondents to characterize themselves using both absolute ratings and ratings relative to peers, whereas the other two items offer brief descriptions of happy and unhappy individuals and ask respondents the extent to which each characterization describes them. The total score is derived by summing the four items. The possible range of the total score is 4–28, with a score of 18 to 22 representing an average range. A higher score reflects a greater happiness.

### Depression and anxiety scale

The symptom levels of depression and anxiety were measured using the patient-reported outcome measurement information system (PROMIS) [44, 45]. PROMIS, which is a set of online measure systems developed by the National Institute of Health (NIH) of the United States (US), evaluates multiple physical and mental conditions, including anger, depression, fatigue, anxiety, depression and physical function measures [44, 45]. Both the depression and the anxiety questionnaires are five-item Likert-type response scales to measure the frequency with which respondents have experienced over the past week. Both of them are universal symptom screening tools rather than disease-specific diagnostic tools. A sample item from the anxiety scale is “I felt worried in the past seven days”. Participants were asked to rate their agreement with 5 answer choices including “Never”, “Rarely”, “Sometimes”, “Often” and “Always” (Cronbach’s α = 0.87). PROMIS has two different but highly comparable scoring options: a short form and a computer adaptive test (CAT) [46]. In the short form option, participants are asked to answer an entire set of questions, while the CAT is a response-based scoring system in which participant’s response to the first item will guide the system’s choice of subsequent items, and the computer will calculate the sum score automatically [46]. We adopted a combination of both options: the participants were asked to finish the online CAT survey, and the score from the CAT report was used for the current analysis. They were also asked to answer a paper-version short form survey (12 items included, Appendix 4–5), to enable us know their response to each specific item. For both depression and anxiety in the CAT, a score of 50 is the average for the general population. A higher score represents a greater level of the symptom being measured.

### Data analyses

The data were analyzed by using SPSS (version 18). Independent t-tests were used to determine whether there were any differences between the pre- and post-test scores on each outcome measure. Estimated means were used to describe the average pre-test and post-test scores on the outcome measures. Regression analysis was also performed to examine the relationships between the intervention and effect measures (Appendix 7). An alpha level of 0.05 was used to determine the statistical significance of all results.

## Results

### Baseline measurement of hope, life satisfaction, subjective happiness, and symptoms of anxiety and depression of the participants

We first estimated the baseline psychological status of the participants. For the hope scale, the sum scores of the participants ranged from 25 to 59 (the full score was 64), with a mean of 42 and standard deviation (SD) of 7.2 (Table 2). As compared with the average value in the general population (48), it was found that the score in the present medical student cohort was lower, but we were not able to make clear whether this comparison was statistically significant. For the life satisfaction scale, the total score of the participants ranged from 10 to 33 (the full score was 35), with an average score of 19 (SD = 5.5) (Table 2), which was very similar to the neutral point of the scale (20). For the subjective happiness scale, similar findings were observed: the participants’ sum score ranged from 13 to 24 (the full score was 28), with a mean of 18 (SD = 2.8; Table 2), and was located in the average range of the general population score (18–22). These findings indicate that the life satisfaction and subjective happiness scales of the participants were in the average range of the general population.

**Table 2:** Pre- and post-test assessment of psychological well-being among the participating students

Measurements	Preliminary set			Validation set		
	Pre-course (***N*** = 61) M (SD)	Post-course (***N*** = 49^a^) M (SD)	***P*** value	Pre-course (***N*** = 52) M (SD)	Post-course (***N*** = 46^b^) M (SD)	***P*** value
Hope	42 (7.2)	58 (8.3)	0.03	43 (8.1)	54 (7.9)	0.04
Life satisfaction	19 (5.5)	24 (6.2)	0.026	18 (5.3)	22 (6.1)	0.03
Subjective happiness	18 (2.8)	26 (3.4)	0.009	20 (3.1)	27 (4.3)	0.01
Depression	66 (4.6)	31 (2.8)	0.003	64 (4.3)	38 (3.1)	0.004
Anxiety	63 (5.2)	32 (3.2)	0.004	61 (4.8)	35 (2.9)	0.002

^a^12 students discontinued the study, ^b^6 students discontinued the study

For the depression, the mean CAT score of the participants was 66 (ranging from 42 to 72, SD 4.6) (Table 2), higher than the average value of 50. By surveying the paper-version short form questionnaire of the participants, it was found that 61.2% (30 of 49) of participants reported that they often felt discouraged about the future, and 41% (20 of 49) reported that they often felt emotionally exhausted (data not shown). For the anxiety, the mean CAT score of the participants was 63 (SD 5.2, Table 2). A total of 57.1% (28 of 49) of the participants reported that they often felt worried, and 30% (15 of 51) reported that many situations made them worry. There were also 51% (25 of 49) of the students reporting trouble in relaxing and 18% (9 of 49) in sleeping (data not shown).

### Intervention effects of the training program

Before analyzing each of the items in the questionnaire, we generally asked participants how they subjectively felt about this course by simply asking “Overall, what do you think of this project?” with a four-item Likert-type scale (No useful at all, A little useful, Useful, Very useful). This survey was also anonymous to enable the respondent to answer as honest as possible. Surprisingly, over 95% of the preliminary participants (*n* = 49), and 87% of the validation participants (*n* = 46) reported that they felt this training course was “useful” or “very useful”.

**Fig. 1:**
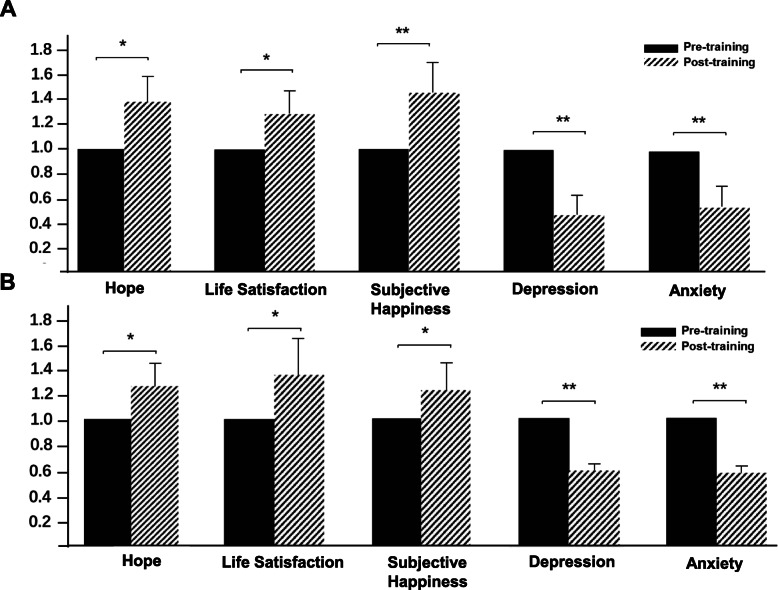
Intervention effects of the positive psychology course. **a** Intervention effects on the preliminary participant cohort. After 8 weeks of positive psychology training, the hope, life satisfaction and subjective happiness scales of the participants were significantly improved, while the depression and anxiety symptoms were relieved. **b** Validation of the intervention effects on an independent cohort of students. The intervention effects of the positive psychology course were validated on an independent cohort of students who enrolled in the course in the year following the above preliminary study. A similar trend was observed. In the figure, the average score of the pre-course group is normalized to 1. *P < 0.05, * *P < 0.01.

We then analyzed whether and how this positive psychology intervention changed the psychological condition of the participants by comparing the pre- and post-course questionnaires. For the hope trait scale, it was found that the mean overall score of the participants increased from 42 in pre-course (*n* = 61) to 58 in post-course (*n* = 49) (by 38%, *P* = 0.03; Fig. 1a, Table 2). For the life satisfaction scale and the subjective happiness scale, a similar trend was observed. The mean score of the life satisfaction scale increased from 19 to 24 (*P* = 0.026), and the mean score of the subjective happiness scale increased from 18 to 26 (*P* = 0.009) (Fig. 1a, Table 2) after the course, respectively. These findings suggest the efficacy of positive psychology training on improving the psychological well-being of Chinese medical students.

For the symptom levels of depression and anxiety, it was found that the respondents had significantly fewer symptoms after the workshop: the mean depression score of the respondents decreased from 66 to 31 (*P* = 0.003), and the mean anxiety score dropped from 63 to 32 (*P* = 0.004) after the training program (Fig. 1a, Table 2). These findings suggest the potential effects of positive psychology in alleviating depressive symptoms among medical students.

### Validation of the intervention effects on an independent cohort of students

To confirm our findings, we validated the above measures in an independent cohort of medical students who took part in the course in the year following the above preliminary study. In consistence with the findings described above, the average overall score of the hope, life satisfaction and subjective happiness scales of this testing cohort of respondents also increased (Table 2, Fig. 1b). The mean overall score of hope increased from 43 to 54 (*P* = 0.04), life satisfaction increased from 18 to 22 (*P* = 0.03), and subjective happiness increased from 20 to 27 (*P* = 0.01) after the training course (Table 2, Fig. 1b). For the depression and anxiety scale, reductions from 64 to 38 and 61 to 35 were observed, respectively (Table 2, Fig. 1b). When asked the general question “Overall, what do you think of this project?” (four-item Likert-type scale: Not useful at all, A little useful, Useful, Very useful), 87% of the participants reported that they felt this training course was “useful” or “very useful”. These findings suggest again the feasibility of applying positive psychology interventions to improve the psychological well-being of medical students in the classroom.

## Discussion

In the present study, we tested and validated the potential effect of a classroom-based positive psychology training course on improving psychological well-being and alleviating depressive symptoms in Chinese medical students. The effects of the intervention seemed promising and encouraging. The hope scale, life satisfaction scale and subjective happiness scale of the participants appeared to improve, while their symptoms of depression and anxiety decreased. These findings suggest the promising effects of positive psychology education on improving the mental well-being of Chinese medical students, and indicate that teaching psychological well-being in school may be feasible and desirable.

### Embedding positive psychology into school education

In this study, we established the training program as an elective course embedded in the regular school curriculum based on two reasons. First, course-based training is more cost-effective and can benefit more students as compared with the traditional one-on-one school counselling service. Moreover, multiple evidences have shown that integrating positive psychology into school education can act not only as an antidote to depression for the general student population, but also as a way to increase their happiness and life satisfaction with diagnostic symptoms [34, 47]. Second, classroom-based training program is more acceptable to the Chinese students than the one-on-one counselling. In contrast to Western population, Chinese people seldom seek help from psychologists even if they are in poor mental state. It was once reported that among people diagnosed with mental illness in China, only less than 10% of them sought help from psychology professionals [48]. The major underlying reason is that in traditional Chinese culture values, seeing a psychologist is a symbol of weakness and vulnerability. This is also why we kept the questionnaire anonymous through the research, although this strategy made the statistical analysis more difficult and less powerful. The promising results observed in the pilot study here suggest that embedding positive psychology into the regular school curriculum to improve students’ psychological status and prevent unprecedented depression and anxiety among general university students may be practical but needs further validation in larger populations with control studies.

### Positive psychology education among medical students

We found that positive psychology intervention may improve the psychological well-being of medical students. Their scores on the hope scale, life satisfaction scale and subjective happiness scale were significantly improved, and at the same time, depression and anxiety symptoms were relieved. These findings indicate the possibility and feasibility of positive psychology intervention in increasing the psychological well-being of medical students. This finding is in agreement with a series of studies that have shown the effectiveness of positive psychology in optimizing health and well-being in general populations, although not in medical professionals [27, 28, 49].

In the medical community, researchers have actually proposed that positive psychological concepts, such as resilience, character strength, and mindfulness, can and should be integrated into clinical practice to help clients alleviate suffering and increase wellbeing [50–53]. For example, Friedman SE et al. discussed how insights from the field of positive psychology and social neuroscience can help healthcare providers and their organizations flourish in both their professional practices and personal lives [51, 52]. However, no direct research evidence has been provided so far. The findings of the current study may therefore provide insights for positive psychology interventions among medical professionals in the future.

### Limitations and future directions

While this study provides promising implications for the application of positive psychology education among Chinese medical students to improve their psychological well-being, the following limitations and weaknesses should be noted.

First, the sample cohorts used in the current study were small, which consisted of only 49 students in the preliminary study and 51 ones in the validation. Moreover, the gender distribution among both participant cohorts was imbalanced, in which female students were more dominant. This may bring bias to the results since females and males have different sensitivities toward psychology. To make the findings more convincing, therefore, a larger participant cohort with balanced gender distribution should be employed in future studies.

Second, there was no follow-up data in the current study, and the duration of the training effect should be estimated in the longer future. It would be interesting to check whether those students who received positive psychology training would demonstrate better psychological well-being after they enter into their clinical professionals. Such an investigation would provide more comprehensive evidence for the effects of positive psychology intervention on improving the psychological well-being of medical students.

Third, there was no control group in the present study, which may reduce the reliability of the findings. Although we performed a paired comparison for each measured item between the pre- and post-course questionnaire, all of the results were assessed at an overall level since the survey was anonymous, which may reduce the statistical power.

Finally, the study design of the current study may bring some biases and exaggerate the positive findings. For example, the project allowed students to enroll and exit voluntarily. It is possible that the students who chose to enroll in the course were more willing to grow emotionally, which may partially or wholly account for the measured improvements. At the same time, the questionnaires from the drop-out students were not included in the analysis. Although their official reason was that they were too busy to follow up, there were possible underlying reasons that they did not think that the training was useful and lost interest in it. These factors together may raise the possibility that a higher positive result was observed.

## Conclusions

In summary, the findings of this preliminary pilot study may provide a basis for classroom-based positive psychology interventions to improve the psychological well-being of medical students in the future. Further assessments on a larger sample cohort may yield more significant and reliable results.

## Supplementary information

**Additional file 1: Appendix 1.** The Trait Hope Scale. **Appendix 2.** The Satisfaction with Life Scale. **Appendix 3.** Subjective Happiness Scale (SHS). **Appendix 4.** Depression Scale. **Appendix 5.** Anxiety Scale. **Appendix 6.** Syllabus of the Course. **Appendix 7.** Regression Analysis of the Data.

## Data Availability

The data that support the findings of this study are freely available to any scientist wishing to use them for non-commercial purposes on request (Contact: Dr. Xiao-Qin Zhang, mczhxq@scut.edu.cn).

## Abbreviations

SCUT: School of Medicine, South China University of Technology
PERMA: Positive emotion, Engagement, Relationship, Meaning, Accomplishment
PROMIS: Patient-Reported Outcome Measurement Information System
SD: Standard Deviation
