# Supplementary material for: Application of a classroom-based positive psychology education course for Chinese medical students to increase their psychological well-being: a pilot study
Source: BMC Med Educ. 2020 Sep 22;20:323. doi: 10.1186/s12909-020-02232-z (PMC7507630; doi:10.1186/s12909-020-02232-z)
Supplement: Supplementary file 1 — Additional file 1: Appendix 1. The Trait Hope Scale. Appendix 2. The Satisfaction with Life Scale. Appendix 3. Subjective Happiness Scale (SHS). Appendix 4. Depression Scale. Appendix 5. Anxiety Scale. Appendix 6. Syllabus of the Course. Appendix 7. Regression Analysis of the Data. [file 12909_2020_2232_MOESM1_ESM.docx]

# Appendix 1

# The Trait Hope Scale

**By Snyder CR, Ph.D.**

Directions: Read each item carefully. Using the scale shown below, please select the number that best describes YOU and put that number in the blank provided.

1. = Definitely False

2. = Mostly False

3. = Somewhat False

4. = Slightly False

5. = Slightly True

6. = Somewhat True

7. = Mostly True

8. = Definitely True

___ 1. I can think of many ways to get out of a jam.

___ 2. I energetically pursue my goals.

___ 3. I feel tired most of the time.

___ 4. There are lots of ways around any problem.

___ 5. I am easily downed in an argument.

___ 6. I can think of many ways to get the things in life that are important to me.

___ 7. I worry about my health.

___ 8. Even when others get discouraged, I know I can find a way to solve the problem.

___ 9. My past experiences have prepared me well for my future.

___10. I’ve been pretty successful in life.

___11. I usually find myself worrying about something.

___12. I meet the goals that I set for myself.

Note. When administering the scale, it is called The Future Scale. The agency subscale score is derived by summing items 2, 9, 10, and 12; the pathway subscale score is derived by adding items 1, 4, 6, and 8. The total Hope Scale score is derived by summing the four agency and the four pathway items.

# Reference:

1. Snyder CR, Harris C, Anderson JR, Holleran SA, Irving LM, Sigmon ST, Yoshinobu L, Gibb J, Langelle C, Harney P: The will and the ways: development and validation of an individual-differences measure of hope. Journal of personality and social psychology. 1991, 60(4):570-585

2. Snyder CR: Hope theory: Rainbows in the mind. Psychol Inq. 2002, 13(4):249-275.

# Appendix 2

# The Satisfaction with Life Scale

**By Ed Diener, Ph.D.**

DIRECTIONS: Below are five statements with which you may agree or disagree. Using the 1-7 scale below, indicate your agreement with each item by placing the appropriate number in the line preceding that item. Please be open and honest in your responding.

1 = Strongly Disagree

2 = Disagree

3 = Slightly Disagree

4 = Neither Agree or Disagree

5 = Slightly Agree

6 = Agree

7 = Strongly Agree

___ 1. In most ways my life is close to my ideal.

___ 2. The conditions of my life are excellent.

___ 3. I am satisfied with life.

___ 4. So far I have gotten the important things I want in life.

___ 5. If I could live my life over, I would change almost nothing.

# Reference:

1. Diener E, Emmons, R. A., Larsen, R. J., & Griffin, S. : The Satisfaction with Life Scale. Journal of Personality Assessment, 49, 71-75. Journal of Personality Assessment 1985, 49:5.

2. Emerson SD, Guhn M, Gadermann AM: Measurement invariance of the Satisfaction with Life Scale: reviewing three decades of research. Quality of Life Research 2017, 26(9):2251-2264.

# Appendix 3

# Subjective Happiness Scale (SHS)

**By Sonja Lyubomirsky, Ph.D.**

For each of the following statements and/or questions, please circle the point on the scale that you feel is most appropriate in describing you.

1. In general, I consider myself:

1 2 3 4 5 6 7

not a very happy person a very happy person

2. Compared to most of my peers, I consider myself:

1 2 3 4 5 6 7

less happy more happy

3. Some people are generally very happy. They enjoy life regardless of what is going on,

getting the most out of everything. To what extent does this characterization describe you?

1 2 3 4 5 6 7

not at all a great deal

4. Some people are generally not very happy. Although they are not depressed, they never

seem as happy as they might be. To what extent does this characterization describe you?

1 2 3 4 5 6 7

not at all a great deal

# Reference:

1. Sonja Lyubomirsky HSL: A measure of subjective happiness: Preliminary reliability and construct validation. Social Indicators Research 1999, 46(2):19.

2. Lyubomirsky S: Why are some people happier than others?: The role of cognitive and motivational processes in well-being. American Psychologist 2001, 56:11.

# Appendix 4

# Depression Scale

**From PROMIS, Patient-Reported Outcomes Measurement Information System, developed by** **the U. S. Department of Health and Human Services.**

Directions: Read each item carefully. Using the scale shown below, please select the number that best describes YOU and put that number in the blank provided.

1. = Never

2. = Rarely

3. = Sometimes

4. = Often

5. = Always

In the past 7 days

___ 1. I felt depressed

___ 2. I felt unhappy

___ 3. I felt sad

___ 4. I felt disappointed in myself

___ 5. I felt pessimistic

___ 6. I felt emotionally exhausted

___ 7. I felt discouraged about the future

___ 8. I felt lonely

___ 9. I felt guilty

___ 10. I had trouble feeling close to people

___ 11. I had trouble making decisions

___ 12. I felt that I was to blame for things

# Reference:

# 1. Hays RD, Bjorner JB, Revicki DA, Spritzer KL, Cella D: Development of physical and mental health summary scores from the patient-reported outcomes measurement information system (PROMIS) global items. Quality of life research: an international journal of quality of life aspects of treatment, care and rehabilitation 2009, 18(7):873-880.

2. Choi SW, Reise SP, Pilkonis PA, Hays RD, Cella D: Efficiency of static and computer adaptive short forms compared to full-length measures of depressive symptoms. Quality of life research : an international journal of quality of life aspects of treatment, care and rehabilitation 2010, 19(1):125-136.

# Appendix 5

# Anxiety Scale

**From PROMIS, Patient-Reported Outcomes Measurement Information System, developed by** **the U. S. Department of Health and Human Services.**

Directions: Read each item carefully. Using the scale shown below, please select the number that best describes YOU and put that number in the blank provided.

1. = Never

2. = Rarely

3. = Sometimes

4. = Often

5. = Always

___ 1. I felt uneasy

___ 2. I felt tense

___ 3. I felt worried

___ 4. I felt upset

___ 5. I had trouble relaxing

___ 6. Many situations made me worry

___ 7. I had difficulty sleeping

___ 8. I had trouble paying attention

___ 9. I felt indecisive

___ 10. I felt nervous

___ 11. I felt anxious

___ 12. I was anxious if my normal routine was disturbed

# Reference:

# 1. Hays RD, Bjorner JB, Revicki DA, Spritzer KL, Cella D: Development of physical and mental health summary scores from the patient-reported outcomes measurement information system (PROMIS) global items. Quality of life research: an international journal of quality of life aspects of treatment, care and rehabilitation 2009, 18(7):873-880.

2. Choi SW, Reise SP, Pilkonis PA, Hays RD, Cella D: Efficiency of static and computer adaptive short forms compared to full-length measures of depressive symptoms. Quality of life research : an international journal of quality of life aspects of treatment, care and rehabilitation 2010, 19(1):125-136.

# Appendix 6

# Syllabus of the Course

| **No.** | **Topic of the workshop** | **Assignments** | **Recommended Reading (Book)** |
| --- | --- | --- | --- |
| Lecture 1 | Why positive psychology | Writing down the expectation on the course, or the problems that wanted to be settled most in class | Flourish  --- by Martin Seligman  (Chinese version) |
| Lecture 2 | Empowerment, and self-management | Setting goals | Be your personal best  ---by Kaifu Lee  (Chinese version) |
| Lecture 3 | Strength and virtue | Writing down character strength | Flourish  ---by Martin Seligman  (Chinese version) |
| Lecture 4 | Extrovert and introvert | Discussion: are you an extrovert or introvert? | Quiet: the power of introverts in a world that can't stop talking  ---by Susan Cain  (Chinese version) |
| Lecture 5 | Happiness, gratitude and forgiveness | Writing down three good things; Gratitude visiting | Authentic happiness  ---by Martin Seligman  (Chinese version) |
| Lecture 6 | Mood and achievement | Seminar: how to be in harmony with the bad mood Positive emotion; growth mindset | The power of now  ----by Eckhart Tolle  (Chinese version) |
| Lecture 7 | Thriving at work: turn work into flow | Discussion: how to achieve flow | Flow  ---by Mihaly Csikszentmihalyi  (Chinese version) |
| Lecture 8 | Work and meaning | Discussion: doctor-patient relationship | PEAK: secrets from the new science of expertise  ---by Anders Ericsson  (Chinese version) |

**Instructor:** Dr. Xiao-Qin Zhang; Teaching assistant: Miss Meng-Die Wang

#

# Appendix 7

# Regression Analysis of the Data

|  |  | **Hope** | **Life satisfaction** | **Subjective happiness** | **Depression** | **Anxiety** |
| --- | --- | --- | --- | --- | --- | --- |
|  | Mean of Slope | 30.69 | 33.76 | 12.48 | -36.59 | -33.57 |
| **Training set** | 95% Confidence Intervals of Slope | 25.04 to 36.35 | 17.45 to 50.08 | 10.57 to 14.38 | -38.14 to -35.05 | -35.39 to -31.74 |
|  | P value | <0.0001 | <0.0001 | <0.0001 | <0.0001 | <0.0001 |
|  |  |  |  |  |  |  |
|  |  |  |  |  |  |  |
|  | Mean of Slope | 28.93 | 31.58 | 13.21 | -33.89 | -34.32 |
| **Validation set** | 95% Confidence Intervals of Slope | 25.03 to 32.84 | 15.27 to 47.9 | 11.3 to 15.13 | -35.44 to -32.35 | -36.14 to-32.49 |
|  | P value | <0.0001 | <0.0001 | <0.0001 | <0.0001 | <0.0001 |
